# Lateral Tibial Plateau Fracture Following Medial Unicompartmental Knee Arthroplasty: A Case Report

**DOI:** 10.7759/cureus.107507

**Published:** 2026-04-21

**Authors:** Tomohito Iga, Takafumi Hiranaka, Koji Okamoto, Takaaki Fujishiro, Motoki Koide

**Affiliations:** 1 Orthodontics, Takatsuki General Hospital, Takatsuki, JPN; 2 Orthopaedic Surgery and Joint Surgery Centre, Takatsuki General Hospital, Takatsuki, JPN

**Keywords:** conservative management, lateral tibial plateau fracture, medial unicompartmental knee arthroplasty (uka), oxford knee, postoperative complications

## Abstract

Medial unicompartmental knee arthroplasty (mUKA) is a well-established surgical option for isolated medial compartment osteoarthritis. While tibial medial plateau fractures are recognized postoperative complications, lateral plateau fractures are exceptionally rare. To our knowledge, this report presents, for the first time, a case of a complete lateral tibial plateau fracture immediately following mUKA.

A 76-year-old man with a 30-year history of left knee pain and a diagnosis of osteoarthritis underwent cementless mUKA (Oxford system). Postoperative radiographs taken on the day after surgery revealed a Schatzker Type I lateral tibial plateau fracture. The articular surface was undisturbed, and the bone fragment remained stable, likely due to fibular support. Conservative treatment was implemented without weight-bearing restrictions. No braces were used, and no rest restrictions were imposed. Bone union was achieved, with no progression of displacement. At one-year follow-up, the patient was pain-free and able to ambulate independently. This case highlights a rare but significant complication: a complete lateral tibial plateau fracture following mUKA. Unlike previously reported cases of lateral insufficiency fractures, this fracture was complete and occurred immediately postoperatively.

The etiology remains unclear, but fibular stabilization notably facilitated successful conservative management without weight-bearing restrictions. The Japan Orthopaedic Association Score before surgery was 60, but improved to 75 one year after surgery. The Knee Society Score before surgery was 60, but improved to 95 one year after surgery. The Oxford Knee score before surgery was 19, but improved to 50 after surgery. This case underscores the importance of recognizing such rare complications and demonstrates that conservative treatment can achieve excellent clinical outcomes in cases of stable fractures.

## Introduction

Medial unicompartmental knee arthroplasty (mUKA) is established as a reliable and durable procedure for relieving pain and improving function in patients with isolated medial compartment osteoarthritis [1]. The benefit of mUKA has been reported to be lower invasiveness, quicker recovery, and lower mortality and systemic complications compared with total knee arthroplasty [2]. However, several specific complications, such as lateral osteoarthritis progression and tibial component subsidence, have been reported after mUKA. Above all, tibial fracture is one of the most serious complications. Fractures of the tibial plateau may occur after mUKA and account for 2% of total UKA failures [3]. Most fractures are reported at the medial tibial plateau, and lateral tibial fractures after mUKA have been seldom reported. Only one report by Tanaka et al. described four cases of insufficiency fractures of the lateral tibial plateau, which were successfully treated conservatively [4]. In this report, we present a case of a complete lateral tibial plateau fracture immediately after mUKA.

## Case presentation

This case report was conducted in accordance with the principles of the Declaration of Helsinki (2024 revision). Written informed consent was obtained from the patient (or their legal guardian) for the publication of this report and any accompanying images. Patient anonymity has been preserved throughout.

**Figure 1:**
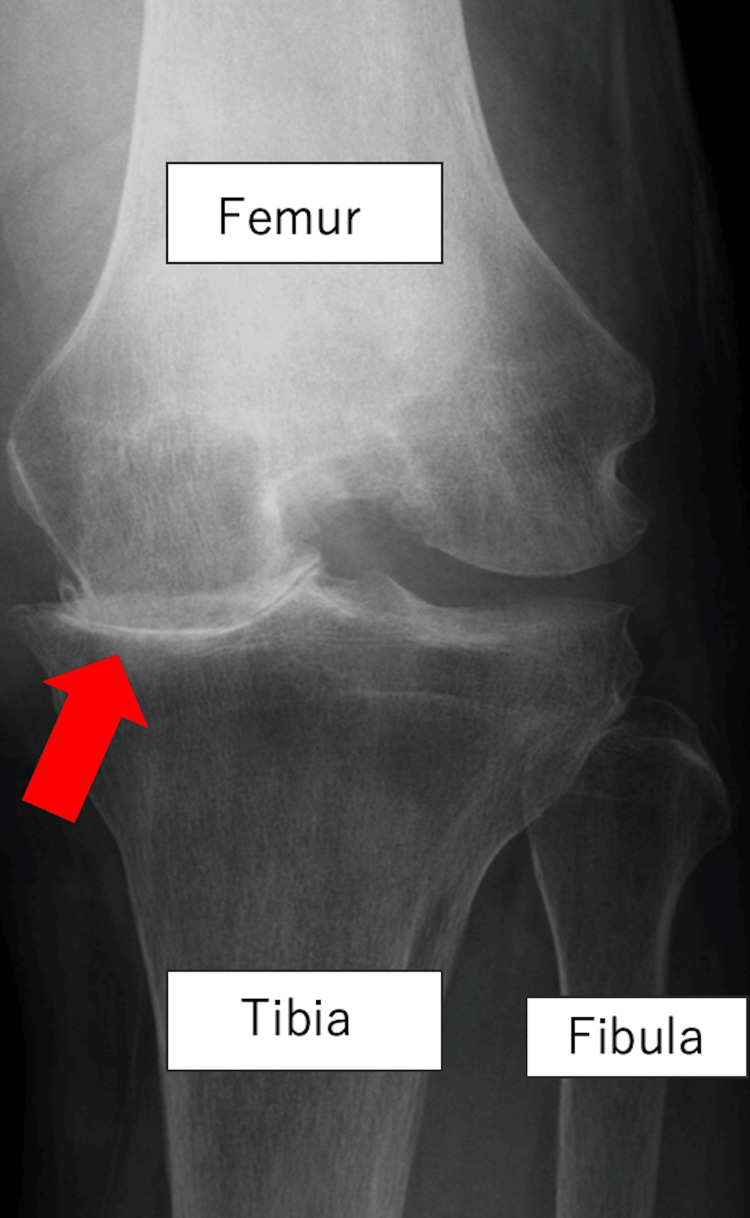
Preoperative X-ray shows the loss of the medial joint space (arrow).

A 76-year-old man had a 30-year history of left knee pain. Initial radiographs at our institution showed the absence of the medial joint space in the left knee (Figure 1). Kellgren-Lawrence's grade was IV. Valgus stress testing revealed correctable varus deformity and preservation of the lateral joint space, and medial tibial thrust was also observed. The patient’s condition was reflected by a Japanese Orthopaedic Association score of 60 points, a Knee Society Score-Knee Objective Score of 60 out of 100, and an Oxford Knee Score of 19 out of 48. Preoperative MRI confirmed continuity of the anterior cruciate ligament with no visible fractures in the lateral plateau. Conservative treatment for osteoarthritis provided insufficient relief, and he was referred to our institution. Therefore, we performed mUKA in this patient. mUKA was performed using a cementless Oxford Partial Knee system (Zimmer Biomet Inc., Warsaw, IN, USA) via the subvastus approach. A size M femoral component and a size D tibial component were implanted with a size 4 mobile bearing. Bone cutting was performed with mild varus alignment using custom instrumentation unique to our institution. The custom instrumentation was used to perform a varus osteotomy, as it has been reported that performing a varus osteotomy reduces the incidence of medial condyle fractures [5].

**Figure 2:**
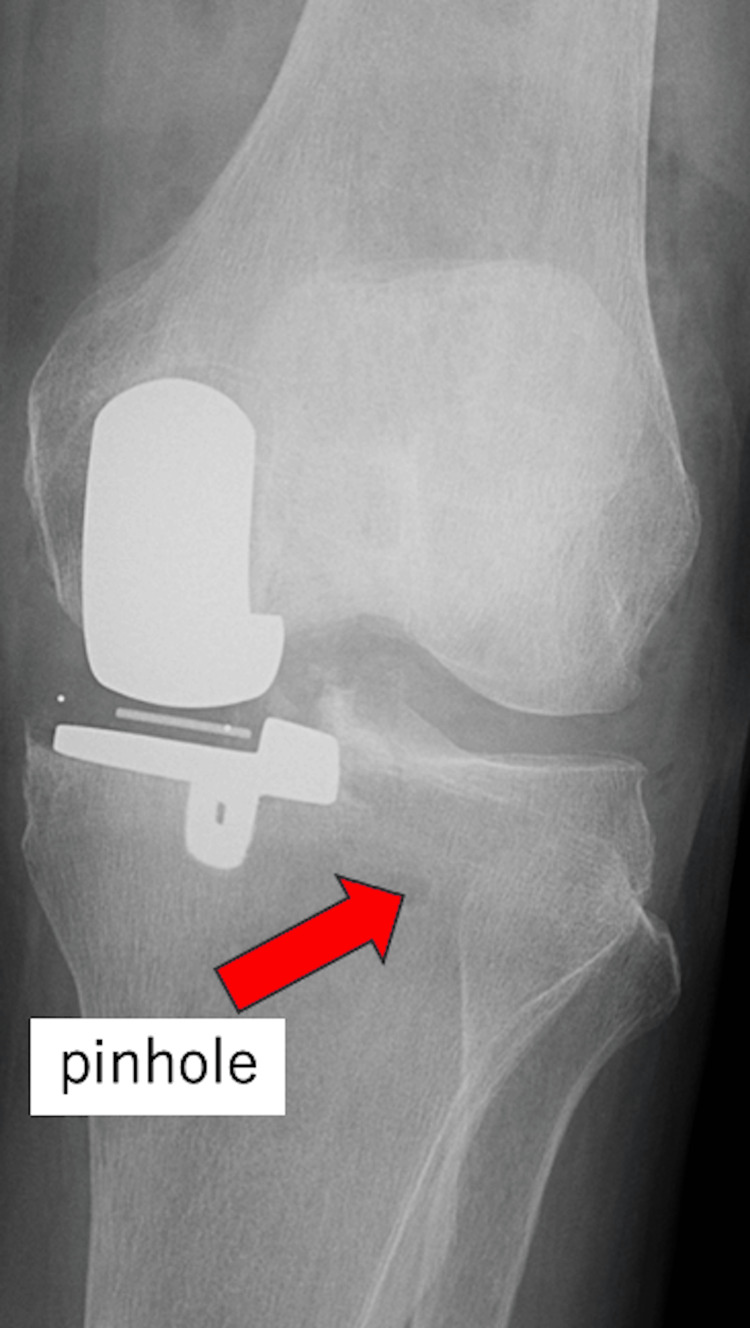
Immediate postoperative X-ray shows no obvious fractures (arrow).

**Figure 3:**
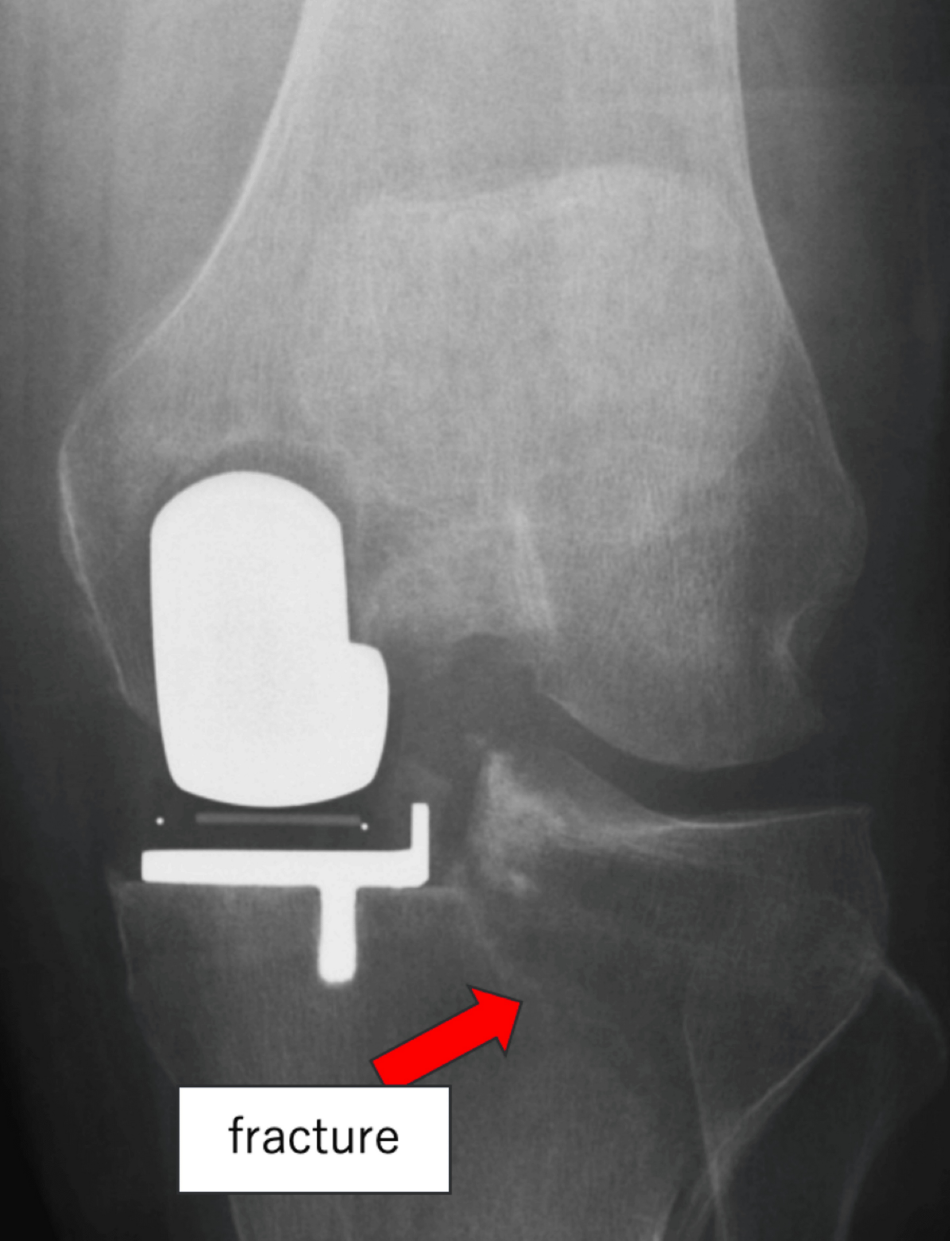
X-ray taken the day after surgery shows a lateral tibial plateau fracture (arrow).

Postoperative radiographs were unremarkable (Figure 2). The patient began weight bearing the day after surgery and was able to walk with a cane. Weight bearing was fairly well tolerated, and there was no increase in pain immediately after weight bearing. However, on the following day, radiographs showed a Schatzker Type I complete lateral tibial plateau fracture (Figure 3).

**Figure 4:**
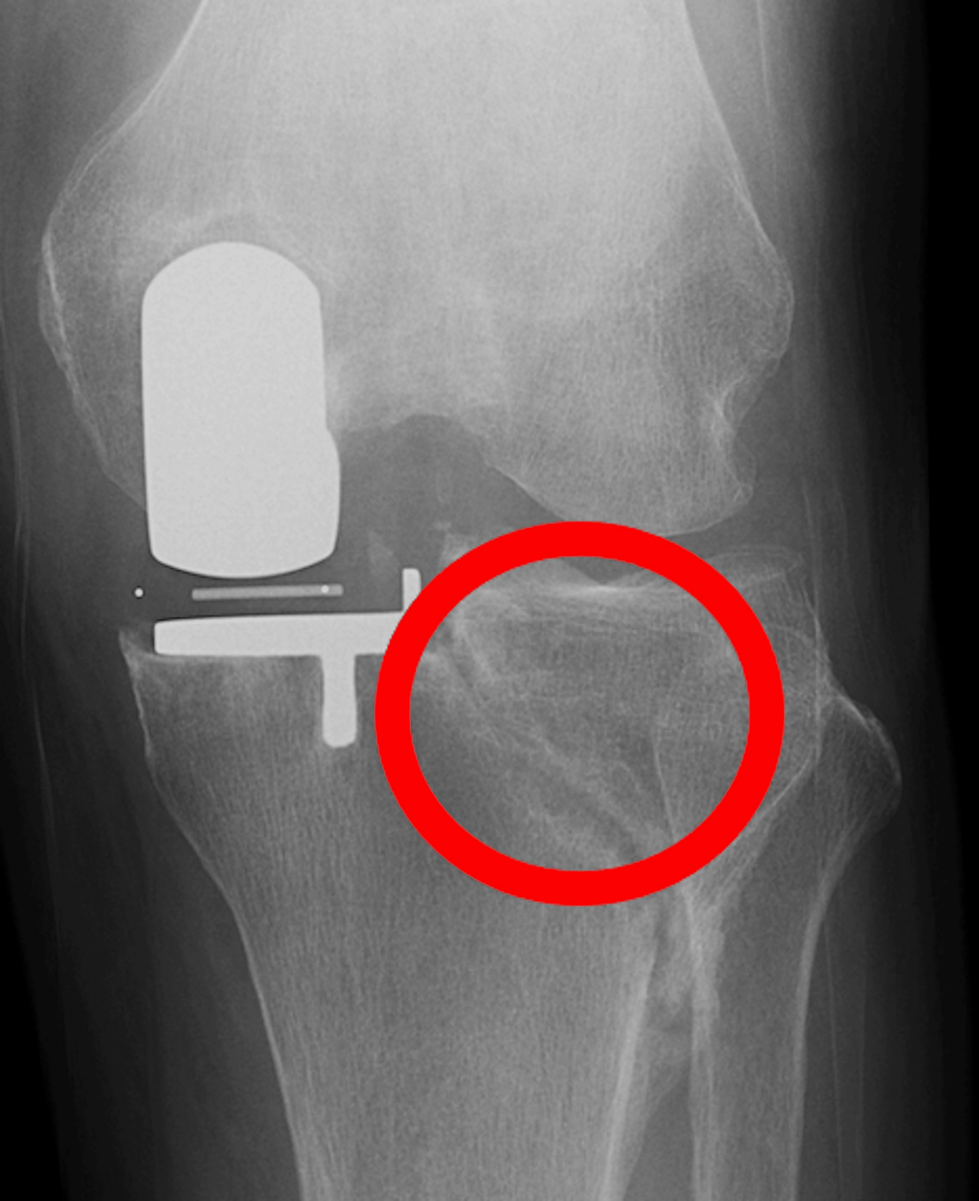
Complete bone union with no displacement is shown on radiographs 6 months postoperatively (circle).

**Figure 5:**
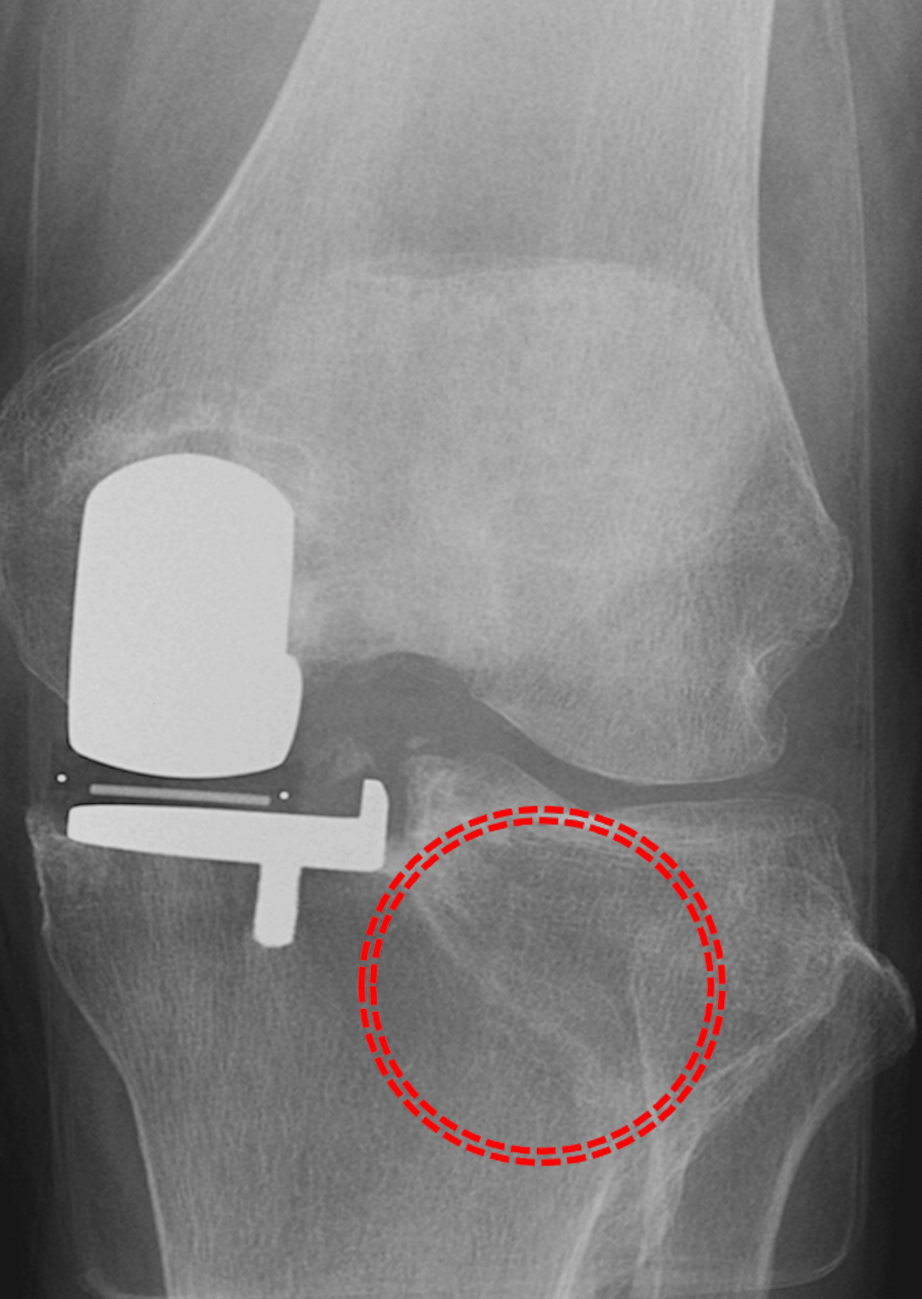
No obvious displacement is observed on the X-ray one year after surgery (dotted circle).

There was no significant joint line displacement on the X-ray, and the patient had no worsening pain. The bone fragment remained stable, and weight-bearing was unproblematic, so no weight-bearing restrictions were imposed. The patient was managed conservatively, and no progression of displacement occurred during hospitalization. He was discharged three weeks postoperatively and then followed regularly as an outpatient. Complete bone union with no displacement was shown on radiographs six months postoperatively (Figure 4). At the one-year follow-up, the patient was pain-free and walking independently (Figure 5).

## Discussion

We report a complete lateral tibial plateau fracture occurring immediately after mUKA. Tibial plateau fractures after mUKA are rare, with reported rates ranging between 0.1% and 8% [6]. Most reported tibial plateau fractures after mUKA involve the medial plateau. A previous report of lateral tibial plateau fractures after mUKA included only insufficiency fractures [4]. To the best of our knowledge, this report is the first documented case of a complete lateral tibial plateau fracture occurring immediately after mUKA.

In this case, no fractures were noted under fluoroscopy immediately after surgery. The day after surgery, there was no apparent increase in pain during initial weight bearing. However, a complete lateral tibial fracture appeared on X-ray the day after surgery. There was no obvious trauma before the fracture was discovered.

The etiology of tibial plateau fracture is typically non-traumatic and tends to be caused by intraoperative tibial micro-damage or repetitive loading during the early postoperative period [7,8]. Risk factors for fractures have been reported to include proximal tibial varus, female sex, and smaller tibial size [9]. This complication can be significantly reduced by performing varus osteotomy [5]. None of these factors applied in this case. Cementless mUKA has also been associated with a higher incidence of tibial plateau fractures than cemented mUKA [7]. In addition, pin holes from tibial cutting guides may serve as stress risers for fractures [1]. In this case, the fracture line traversed the pin holes. Preoperative radiographs also showed medial tibial thrust, which may have contributed to excessive stress on the lateral tibial plateau, resulting in fracture.

In this case, conservative treatment without fixation or weight-bearing restrictions resulted in successful bone healing despite the complete nature of the fracture. Three treatment options are typically considered for tibial plateau fractures after mUKA: conservative management, open reduction and internal fixation (ORIF), or conversion to total knee arthroplasty [6]. Conservative treatment is appropriate for stable fractures with minimal displacement, while ORIF is indicated for larger, displaced fragments. Total knee arthroplasty may be considered for unstable implants or fractures for which ORIF might be unsuitable [6]. Since weight-bearing can cause displacement, internal fixation or conversion to TKA is necessary in medial plateau fractures. However, this case involved a lateral tibial plateau fracture. The bone fragment is also supported by the fibula and surrounding ligaments. Furthermore, quadriceps activity creates a compressive force between the bone fragments. For these reasons, we chose conservative treatment. In this case, the fracture healed with conservative treatment; however, we believe that careful follow-up is necessary, and that additional internal fixation may be required in some cases.

Conservatively treated lateral insufficiency fractures have been reported in the literature, but not cases of conservative treatment of complete fractures [4]. The stability of the lateral tibial fragment, which is anchored by the anterior cruciate ligament, posterior cruciate ligament, and fibula, may explain the success of conservative treatment in this case. So, we recommend conservative treatment in similar cases.

## Conclusions

To our knowledge, we report the first documented case of a Schatzker Type I complete lateral tibial plateau fracture occurring immediately after mUKA. Despite its unknown etiology, conservative treatment without weight-bearing restrictions resulted in successful bone healing and excellent clinical outcomes. There are many case reports of tibial plateau fractures after mUKA, but most of these fractures involve the medial plateau. Therefore, the treatment of lateral tibial fractures after mUKA is not well established. This case may be helpful in guiding the management of similar fracture cases after mUKA in the future.

## References

[1] Chalmers BP, Mehrotra KG, Sierra RJ, Pagnano MW, Taunton MJ, Abdel MP (2018). Reliable outcomes and survivorship of unicompartmental knee arthroplasty for isolated compartment osteonecrosis. Bone Joint J.

[2] Fiocchi A, Condello V, Madonna V, Bonomo M, Zorzi C (2017). Medial vs lateral unicompartmental knee arthrroplasty: clinical results. Acta Biomed.

[3] Thoreau L, Morcillo Marfil D, Thienpont E (2022). Periprosthetic fractures after medial unicompartmental knee arthroplasty: a narrative review. Arch Orthop Trauma Surg.

[4] Tanaka A, Hiranaka T, Fujishiro T, Koide M, Okamoto K (2024). Tibial lateral condyle fracture after cementless Oxford unicompartmental knee arthroplasty (UKA): a report of four cases. Cureus.

[5] Suda Y, Hiranaka T, Kamenaga T, Koide M, Fujishiro T, Okamoto K, Matsumoto T (2022). Varus placement of the tibial component of Oxford unicompartmental knee arthroplasty decreases the risk of postoperative tibial fracture. Bone Joint J.

[6] Wood MJ, Al-Jabri T, Maniar AR, Stelzhammer T, Lanting B, Giannoudis PV (2024). Periprosthetic tibial fracture as a complication of unicompartmental knee arthroplasty: current insights. Injury.

[7] Burger JA, Jager T, Dooley MS, Zuiderbaan HA, Kerkhoffs GM, Pearle AD (2022). Comparable incidence of periprosthetic tibial fractures in cementless and cemented unicompartmental knee arthroplasty: a systematic review and meta-analysis. Knee Surg Sports Traumatol Arthrosc.

[8] Mohammad HR, Barker K, Judge A, Murray DW (2023). A comparison of the periprosthetic fracture rate of unicompartmental and total knee replacements. J Bone Joint Surg Am.

[9] Hiranaka T, Yoshikawa R, Yoshida K (2020). Tibial shape and size predicts the risk of tibial plateau fracture after cementless unicompartmental knee arthroplasty in Japanese patients. Bone Joint J.

